# α7 Nicotinic acetylcholine receptor: a key receptor in the cholinergic anti-inflammatory pathway exerting an antidepressant effect

**DOI:** 10.1186/s12974-023-02768-z

**Published:** 2023-03-27

**Authors:** Huiyang Liu, Xiaomei Zhang, Peng Shi, Jiyuan Yuan, Qiang Jia, Chao Pi, Tao Chen, Linjin Xiong, Jinglin Chen, Jia Tang, Ruxu Yue, Zerong Liu, Hongping Shen, Ying Zuo, Yumeng Wei, Ling Zhao

**Affiliations:** 1 Key Laboratory of Medical Electrophysiology, Ministry of Education, School of Pharmacy of Southwest Medical University, Luzhou, 646000 People’s Republic of China; 2 Key Laboratory of Medical Electrophysiology, Ministry of Education, The Affiliated Traditional Chinese Medicine Hospital of Southwest Medical University, No. 182, Chunhui Road, Longmatan District, Luzhou, 646000 Sichuan People’s Republic of China; 3 Central Nervous System Drug Key Laboratory of Sichuan Province, School of Pharmacy of Southwest Medical University, Luzhou, 646000 Sichuan People’s Republic of China; 4 Luzhou Key Laboratory of Traditional Chinese Medicine for Chronic Diseases Jointly Built by Sichuan and Chongqing, The Affiliated Traditional Chinese Medicine Hospital of Southwest Medical University, Luzhou, 646000 Sichuan People’s Republic of China; 5 Luzhou Key Laboratory of Traditional Chinese Medicine for Chronic Diseases Jointly Built by Sichuan and Chongqing, Institute of Medicinal Chemistry of Chinese Medicine, Chongqing Academy of Chinese Materia Medica, Chongqing, 400065 People’s Republic of China; 6 Clinical Trial Center, The Affiliated Traditional Chinese Medicine Hospital of Southwest Medical University, Luzhou, 646000 Sichuan People’s Republic of China; 7 Ethics Committee Office, The Affiliated Traditional Chinese Medicine Hospital of Southwest Medical University, Luzhou, 646000 Sichuan China; 8 Central Nervous System Drug Key Laboratory of Sichuan Province, Sichuan Credit Pharmaceutical CO., Ltd., Luzhou, 646000 Sichuan China; 9 Key Laboratory of Biorheological Science and Technology, Ministry of Education, College of Bioengineering, Chongqing University, Chongqing, 400030 China; 10 Department of Comprehensive Medicine, The Affiliated Traditional Chinese Medicine Hospital of Southwest Medical University, No. 182, Chunhui Road, Longmatan District, Luzhou, 646000 Sichuan China

**Keywords:** Depression, Inflammation, α7 nAChR

## Abstract

Depression is a common mental illness, which is related to monoamine neurotransmitters and the dysfunction of the cholinergic, immune, glutamatergic, and neuroendocrine systems. The hypothesis of monoamine neurotransmitters is one of the commonly recognized pathogenic mechanisms of depression; however, the drugs designed based on this hypothesis have not achieved good clinical results. A recent study demonstrated that depression and inflammation were strongly correlated, and the activation of alpha7 nicotinic acetylcholine receptor (α7 nAChR)-mediated cholinergic anti-inflammatory pathway (CAP) in the cholinergic system exhibited good therapeutic effects against depression. Therefore, anti-inflammation might be a potential direction for the treatment of depression. Moreover, it is also necessary to further reveal the key role of inflammation and α7 nAChR in the pathogenesis of depression. This review focused on the correlations between inflammation and depression as well-discussed the crucial role of α7 nAChR in the CAP.

## Introduction

Depression is a common mental illness, which is clinically manifested as persistent depressed mood, loss of interest, and cognitive dysfunction, and the disease burden caused by depression ranks first among all mental illnesses [1, 2]. It has been predicted that by 2030, depression will surpass cardiovascular and cerebrovascular diseases and become the first largest disease, causing human death and disability [3]. Numerous studies showed that the pathogenesis of depression might be related to the low levels of monoamine neurotransmitters and the dysfunction of multiple systems in the body, such as the cholinergic, immune, glutamatergic, and neuroendocrine systems [4–15]. At present, the hypothesis of monoamine neurotransmitters is a commonly recognized pathogenic mechanism of depression [4]. Therefore, the pharmacological effects of currently available antidepressants, such as fluoxetine and venlafaxine, are mainly exerted by blocking the reuptake of monoamine neurotransmitters and increasing the levels of 5-hydroxytryptamine (5-HT), dopamine (DA), and norepinephrine (NE) in the synaptic cleft. However, the results of most double-blind trials showed that the disadvantages of these drugs were that they usually showed effects after 2–4 weeks of administration, and about 40% of patients with depression were non-responsive to these drugs [16, 17]. This suggested that the potential pathophysiology of depression was not well-understood, and the antidepressant therapies based on the hypothesis of monoamine neurotransmitters had certain limitations. Therefore, a more comprehensive elucidation of the pathogenesis of depression is urgently needed to develop more effective antidepressant drugs.

The cholinergic system is made up of enzymes-active substances (involved in manufacturing acetylcholine, ACh), cholinergic neurons or histiocytes (release ACh), and receptors (bind to ACh). The cholinergic system can organize into a network in the body, perform various complex functions [18], and take part in the regulation of learning, cognition, memory, and emotion [19–21]. As early as 1972, Davidson et al. [22] put forward the cholinergic hypothesis of depression and suggested that the occurrence of depression was closely related to the enhancement of cholinergic substance activity in the brain. For example, studies showed that depressive behavior could be induced by elevating central choline and ACh levels or blocking the activity of acetylcholinesterase (AchE) [7–9]. On the contrary, increasing the AchE activity in the hippocampus could reverse depression- and anxiety-like behavior in mice caused by physostigmine, an AchE inhibitor [23].

The functions performed by the cholinergic system are mediated by the cholinergic receptors, including the muscarinic ACh receptor and nicotinic ACh receptor [9]. Interestingly, in recent years, increasing studies have found that the activation of the CAP can exert an antidepressant effect, which is inconsistent with the above-mentioned cholinergic hypothesis for depression [24–26]. CAP is a neuroimmune regulatory pathway; when the central nervous system (CNS) is stimulated by immunity, it can activate the vagus nerve and urge the nerve endings to release ACh. The released ACh can activate α7 nAChR on the surface of various immune cells, such as macrophages and microglia, down-regulate the release of related inflammatory factors, and finally inhibit peripheral and central inflammatory reactions [27]. Therefore, the activation of α7 nAChR-mediated CAP might be a promising direction in antidepressant therapies. The correlations between depression and inflammation, which is mediated by α7 nAChR, should be urgently explored to identify novel antidepressant drugs. This review focused on these correlations and the crucial role of α7 nAChR in the CAP.

## Relationship between inflammation and depression

### Evidence of inflammation associated with depression

The correlation between the nervous and immune systems has been widely studied [24]. Numerous recent studies suggested that inflammation was closely related to the pathogenesis of some nervous system diseases, such as depression [10, 11]. For instance, a meta-analysis, including a series of clinical data, showed a correlation between depression and inflammation among children, adolescents, and adults [28], such as the elevated levels of C-reactive protein and interleukin-6 (IL-6) [29–31]. Other studies showed that the levels of proinflammatory cytokines in the peripheral nervous system and CNS tissue of patients with depression were higher as compared to those in healthy subjects [32], and the brain tissue obtained from the victims of depressive suicide exhibited elevated expression levels of interleukin-1beta (IL-1β), IL-6, and tumor necrosis factor (TNF) [33, 34]. In addition, the results of several clinical studies showed that the anti-inflammatory treatment was used to alleviate depression symptoms [35, 36]. For instance, the brain of depression animal models showed high levels of proinflammatory cytokines, and the intraventricular infusion of anti-inflammatory cytokine interleukin-4 (IL-4) might show antidepressant benefits by modifying central neurotransmitters [12, 37, 38]. With the deepening of research, researchers have gradually found that cerebral neuroinflammation is involved in the occurrence of depression, and peripheral immune factors serve as one of the trigger factors of cerebral neuroinflammation [39].

### The occurrence and development of inflammation in depression: from peripheral inflammation to cerebral neuroinflammation

Inflammation is an immune response during infection or trauma. It is the coordinated activation of inflammatory cytokines-mediated signal cascade and is considered a healthy defense mechanism of the body [40–42]. Normal inflammation is necessary for the damages caused by infection, trauma, or neurodegenerative diseases and neuritis is also a method of restoring neural homeostasis. However, when the inflammatory mediators fail to inhibit the pro-inflammatory immune response, a persistent inflammatory state appears, damaging the neurons and the body. Therefore, it is necessary to control inflammation in a certain range to prevent its excessive production [43]. It has been reported that the structural and functional abnormalities of the enteric nervous system and the imbalance of intestinal flora can destroy the integrity of the intestinal barrier, which leads to the leaking of intestinal contents and/or inflammatory mediators into the peripheral blood circulation and triggers of excessive immune response [39, 44].

In peripheral inflammation, Toll-like receptors (TLRs), present on the surface of macrophages and dendritic cells (DCs) of the innate immune system, are recognized by the pathogen-associated molecular pattern (PAMP) or damage-associated molecular pattern (DAMP). This leads to increasing the local and systemic inflammatory activities [45], including triggering the inflammatory signal pathways, such as nuclear factors-kappa B (NF-κB) and mitogen-activated protein kinase (MAPK) signaling pathways, which lead to the overproduction of TNF, interleukin-1 (IL-1), IL-6, and interleukin-18 (IL-18) [46, 47]. These cellular signal transduction mediators can also communicate with the components of the adaptive immune system, such as T cells and B cells [48, 49]. In addition, cyclooxygenase activity can be enhanced by inflammatory cytokines, leading to an increase in prostaglandins levels [48], which further increases inflammation, forming a vicious circle [45]. As a result, the activation of the peripheral immune system can significantly increase proinflammatory cytokine levels.

Due to the existence of blood–brain barrier (BBB), the brain has been traditionally considered as an immune-privileged site; however, numerous studies have shown that peripheral inflammatory cytokines can enter the CNS via cellular, humoral, and neural pathways [46, 50]. BBB is mainly composed of endothelial cells and astrocytes of the capillary wall, and the destruction or absence of either component can affect its function and increase the probability of CNS inflammation [51]. For example, inflammatory factors, including tumor necrosis factor alpha (TNF-α), IL-1β, and IL-6, could impede the tight junctions of endothelial cells in the BBB in the animal models of inflammation [52, 53], increasing the permeability of the BBB, and make the entry of peripheral inflammatory cytokines and immune cells into the brain easier [54]. Furthermore, TNF-α and IL-1β receptors are expressed on endothelial cells, and their activation by inflammatory factors leads to the synthesis of nitric oxide (NO) and prostaglandins in the brain [55]; this further activates microglia and astrocytes, leading to neuroinflammation [50]. Microglia, known as resident immune cells in the CNS, controls the homeostasis of the CNS internal environment and can be divided into basal state microglia, inflammatory state microglia, and anti-inflammatory state microglia [56]. The activation of microglia induces the polarization of basal state microglia into inflammatory state microglia, which is closely associated with the activation of the NF-κB signaling pathway and NOD-like receptor protein 3 (NLRP3) inflammasome [57–59]. Microglia Kv1.3 channels have a crucial role in the activation of NLRP3 inflammasome and neuroinflammation. Kv1.3 is a delayed rectifier voltage-gated K channel, which is widely expressed in the CNS [60]. For example, Di Lucente et al. showed that microglia activation and IL-1β production could be prevented by Kv1.3 knockdown [61]. Moreover, in addition to microglia, astrocytes also play an important role in the immune system of the brain [62]. For example, astrocytes, a BBB component, are involved in regulating BBB permeability, synaptic transmission, and secretion of brain-derived neurotrophic factor (BDNF) [62, 63]. In addition, the release of proinflammatory cytokines from microglia can also activate astrocytes, causing secondary inflammatory reactions, and producing more inflammatory factors, thereby aggravating neurotoxicity, impairing neurogenesis and synaptic plasticity, and ultimately triggering depression and other related psychiatric diseases [57].

It is worth noting that the pathological changes of systemic inflammation-induced neuroinflammation are usually limited and are mainly concentrated in the cortex, hippocampus, amygdala, and other brain regions, which might be due to the diversity of neurons and glial cells in different brain regions [51, 64, 65]. Recently, Wang et al. [66] showed that the release of proinflammatory cytokines was positively correlated with the degree of connexin (Cx) 43 ubiquitination. The gap junction channel of Cx facilitates the communication between neighboring cells, and the role of glial cells in regulating neuroinflammation is mainly based on this function of Cx, which is lost upon the ubiquitination of Cx [67]. Therefore, repairing neuroinflammatory processes might inhibit the ubiquitination of Cx43, which was consistent with the experimental results of Huang et al. and Wang et al. [68, 69].

### Other causes of cerebral neuroinflammation

Cerebral neuroinflammation, in addition to being associated with peripheral immunity, is also related to oxidative stress (OS) [70, 71], mitochondrial dysfunction [72, 73], energy metabolism disorders, nitroenergy system [74, 75], eating habits [76, 77], sleep quality [78], etc., to some extent.

OS is a series of highly reactive cytotoxic events induced by reactive oxygen species (ROS) [70]. ROS can destroy sensitive cellular target compounds, such as lipids, proteins, and DNA. When OS occurs, antioxidants in the body deplete, such as a reduction in glutathione (GSH), leading to the excessive accumulation of ROS. Due to the rich lipid contents in the membrane of brain cells, excessive ROS destroys the structure and function of the phospholipid bilayer of brain cells through lipid peroxide, alters the permeability of the BBB, and ultimately exacerbates neuroinflammation [71]. Therefore, antidepressant effects could theoretically be exerted by reducing OS and neuroinflammatory responses; this is in agreement with the reports of the study by Nouri et al. [79] and Mozafari et al. [73].

A mitochondrion is a key organelle that provides energy for cells and body and can regulate various cellular processes [80]. Similarly, triggering OS leads to mitochondrial dysfunction as well as deficiency in cell energy, which further aggravates mitochondrial damage, forming a vicious circle [72]. The resulting multiple DAMP, such as ROS and lipid oxide, can activate inflammatory signals by activating TLRs and different mechanisms, such as inflammasome formation, which can also change the permeability of the BBB and eventually exacerbate neuroinflammation [73].

l-Arginine can produce NO in brain under the action of nitric oxide synthase (NOS). This enzyme family consists of three subtypes, including inducible NOS (iNOS), endothelial NOS, and neuronal NOS [74]. A study by Beheshti et al. [75] has shown that NO is an activator of neuroinflammatory response. Therefore, nitrergic system are also related to neuroinflammation and play an antidepressant role by reducing the NO level in the body, which leads to the inhibition of NOS; this was consistent with the report of a study by Lorigooini et al. [81]. In addition, a study by Haj-Mirzaian et al. [74] showed that iNOS played a more prominent role in the depression-like behavior induced by amphetamine withdrawal.

### Pathways of neuroinflammation affecting depression

**Fig. 1:**
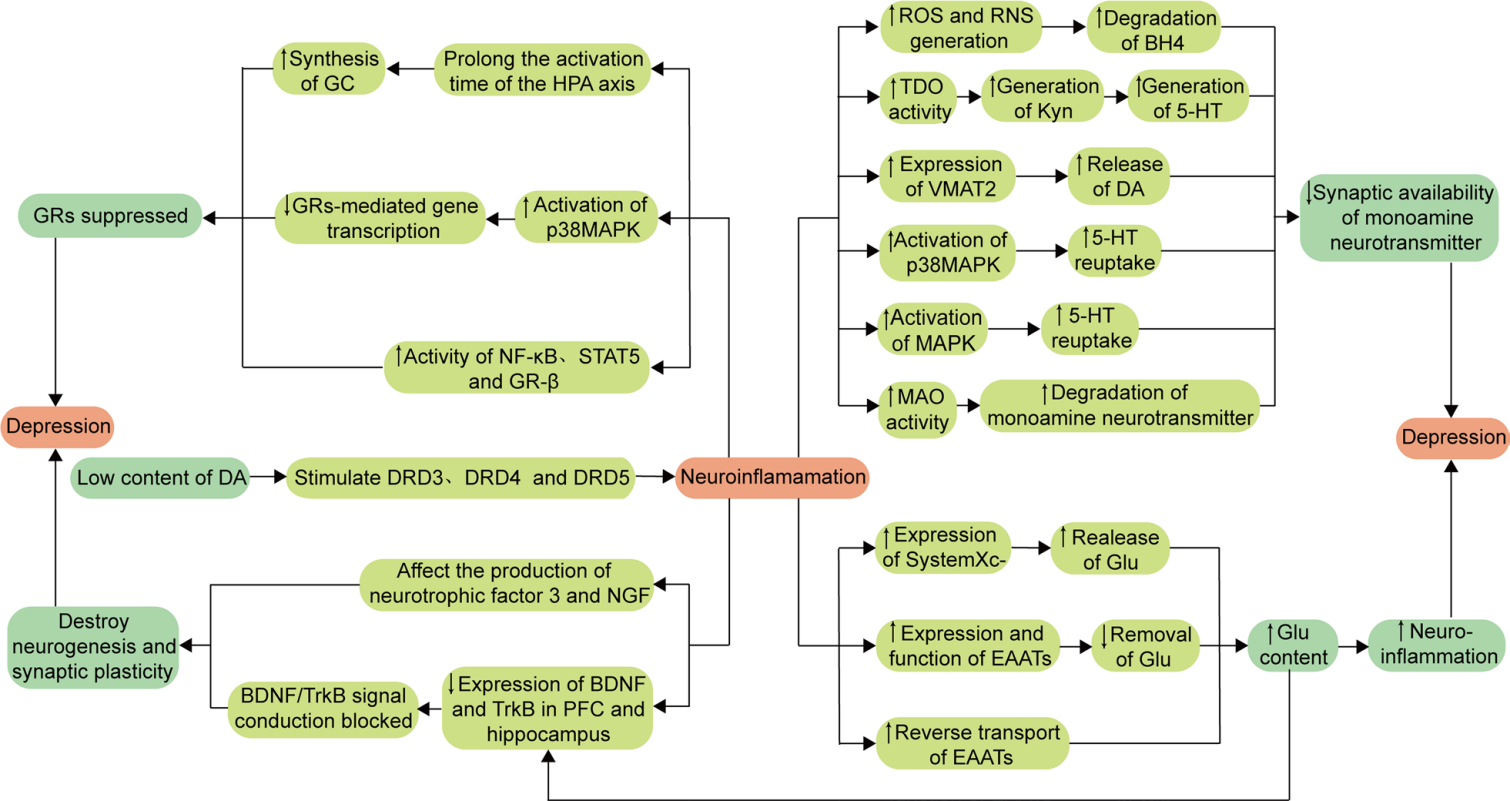
Role of neuroinflammation in the pathogenesis of depression. Low levels of DA can promote the inflammatory reaction, and the produced inflammatory cytokines can increase the probability of depression by reducing the prominent availability of neurotransmitters, increasing neurotoxicity, inhibiting GRs activity, and destroying neurogenesis and synaptic plasticity. ↑: upregulate; ↓: downregulate; *ROS* reactive oxygen species, *RNS* reactive nitrogen species, *BH4* tetrahydrobiopterin, *TDO* tryptophan 2,3-dioxygenase, *Kyn* kynurenine, *5-HT* 5-hydroxytryptamine, *VMAT2* vesicle monoamine transporter 2, *DA* dopamine, *p38MAPK* p38 mitogen-activated protein kinase, *MAPK* mitogen-activated protein kinase, *MAO* monoamine oxidase, *DRD3* dopamine receptors 3, *DRD4* dopamine receptors 4, *DRD5* dopamine receptors 5, *SystemXc−* Cystine/Glu reverse transporter system, *Glu* glutamate, *EAATS* excitatory amino acid reuptake transporters, *HPA* hypothalamus pituitary adrenal, *GC* glucocorticoid, *GRs* glucocorticoid receptors, *NF-κB* nuclear factors-kappa B, *STAT5* signal transducer and activator of transcription 5, *NGF* nerve growth factor, *BDNF* brain-derived neurotrophic factor, *TrkB* tropomyosin receptor kinase B, *PFC* prefrontal cortex

As mentioned above, the overactivation of microglia and astrocytes in the brain results in producing a large number of inflammatory mediators, thereby aggravating neuroinflammation. These inflammatory mediators and downstream signaling pathways can trigger depression by affecting monoamine neurotransmitters, glutamic acid (Glu), the hypothalamus pituitary adrenal (HPA) axis, and neurotrophic factors (NTF) in the body (Fig. 1) [12, 15, 34, 37, 38, 82–87].

#### Effect of neuroinflammation on monoamine neurotransmitters

There is growing evidence, suggesting that inflammatory cytokines can affect the synaptic availability of monoamine neurotransmitters through mechanisms, involving inflammatory cytokines in modulating the synthesis, release, reuptake, and degradation of 5-HT, DA, and NE [34, 82]. This section focuses on the effects of inflammation on the synaptic availability of 5-HT.

Tetrahydrobiopterin (BH4) is a crucial cofactor of tryptophan (Trp), tyrosine, and phenylalanine hydroxylases, which are necessary for the production of 5-HT, DA, and NE. Inflammatory cytokines can exacerbate OS by promoting the formation of ROS and reactive nitrogen species, thereby inducing the degradation of BH4 and reducing the production of 5-HT, DA, and NE. 5-HT is a Trp metabolite, which is an essential amino acid with the lowest levels among the eight essential amino acids; therefore, Trp can easily become deficient in a malnutrition state [88]. Only 1% of the dietary intake of Trp is involved in the biosynthesis of protein, and the majority of the remaining Trp is converted to some bioactive metabolites via the indole, 5-HT, and kynurenine (Kyn) signaling pathways [89]. Through the 5-HT signaling pathway, Trp is converted to 5-hydroxytryptophan by Trp hydroxylase 1 or 2 and then decarboxylated by aromatic acid decarboxylase, forming 5-HT. Then, 5-HT is metabolized by monoamine oxidase (MAO), producing 5-hydroxyindoleacetic acid [90]. About 95% of free Trp is metabolized through the Kyn pathway. In this pathway, Trp, in a rate-limiting step, is first converted to Kyn by indoleamine 2,3-dioxygenase 1 and 2 (IDO1 and IDO2) and tryptophan 2,3-dioxygenase (TDO). Kyn has two major metabolic branches. (1) Kyn is preferentially converted to 3-hydroxykynurenine (3‑HK) or anthranilic acid, catalyzed by kynurenine mono‑oxygenase and kynureninase (Kynu), respectively. The 3-HK is then converted to 3-hydroxy anthranilic acid (3-HAA) and xanthurenic acid by Kynu and kynurenine aminotransferase (KAT), respectively, while 3-HAA is metabolized into picolinic acid and quinolinic acid (QA) by α-amino-β-carboxymuconate-ε-semialdehyde decarboxylase and non-enzymatic conversion, respectively. (2) The remaining Kyn is metabolized by the enzymatic action of KAT into kynurenic acid (Kyna) [91, 92]. Studies showed that inflammatory mediators, such as IL-1, TNF-α, and interferon-γ, could induce the hyperactivation of IDO, which caused more Trp metabolism through the Kyn pathway, thereby decreasing the 5-HT level in the brain [93–95]. According to studies, the Kyn pathway can produce 3-HK and QA, both of which are neurotoxic. For instance, QA cannot cross the BBB, acting as an agonist of the *N*-methyl-d-aspartic acid receptor (NMDAR), while the massive accumulation of QA in the CNS can promote the overactivation of NMDAR in neurons, including those in the striatum and hippocampus [96], thereby increasing the release of Glu and apoptosis rate of neurons [97]. In addition, Kyna can inhibit the release of dopamine, resulting in cognitive impairment [98, 99]. Interestingly, Kyna is also considered a neuroprotective metabolite, acting as an antagonist of α7 nAChR and NMDAR to reduce the release of Glu [100].

DA entry into the vesicle is mediated by monoamine transporter protein 2 (VMAT2) and subsequent release into the synapse. Studies have shown that pro-inflammatory cytokines can down-regulate the expression of VMAT2, thereby interfering with DA release and reducing the level of DA in synaptic cleft, such as IL-1β and TNF-α [101]. Monoamine neurotransmitter transport proteins translocate monoamine neurotransmitters released in the synaptic cleft back to the presynaptic membrane, decreasing synaptic cleft availability [102]. Proinflammatory mediators can regulate the expression and function of monoamine neurotransmitter transporter proteins, thereby altering their reuptake process [36]. For example, proinflammatory cytokines can trigger the p38MAPK signaling pathway, enhancing the expression and activity of 5-HT transporter proteins and decreasing the availability of 5-HT in the synaptic cleft. In addition, the activation of the MAPK signaling pathway can also increase the activity of DA transporter proteins and ultimately reduce the DA levels in the synaptic cleft [103]. Studies have shown that inflammation plays a key role in the degradation of monoamine neurotransmitters by MAO. For example, after lipopolysaccharide administration, the low levels of NE in the hippocampus and elevated levels of an NE metabolite 3-methoxy-4-hydroxyphenylglycol might be associated with elevated monoamine oxidase activity [104].

Notably, some monoamine neurotransmitters, such as dopamine, also play an essential role in regulating the brain’s immune responses by affecting inflammatory responses [105–107]. Kv1.3-induced K^+^ efflux can cause the activation of NLRP3 inflammasome and local fluctuations in extracellular K^+^ concentration, promoting the release of dopamine from proximal dopaminergic neurons [60]. The high levels of dopamine, ranging from 1 to 10 μM, in vivo binding to low-affinity dopamine receptors (DRD1 and DRD2) on microglia could exert anti-inflammatory effects by modulating the proinflammatory renin–angiotensin system [108, 109], and DRD1 could mediate the autophagic degradation of NLRP3 protein. The low levels of dopamine in the body, ranging from 20 to 500 nM, could selectively stimulate the high-affinity dopamine receptors (DRD3, DRD4, and DRD5), thereby inducing inflammatory responses [110].

#### Effect of neuroinflammation on the glutamatergic system

Glu is one of the most prominent excitatory neurotransmitters in the nervous system, acting on Glu receptors and transmitting excitatory signals. There are two types of Glu receptors: (1) ionic receptors, including NMDAR, α-amino-3-hydroxy-5-methyl-4-isoxazole propionic acid receptor, and kainic acid receptor, which mediate the rapid action of postsynaptic potential; and (2) metabolic receptor, which mediates the slow-acting neurotransmission and plays an important role in cellular metabolism [87, 111]. The different Glu-activated receptors can regulate neuronal growth, migration, apoptosis, and synaptogenesis [112]. Therefore, the Glu system might play a crucial role in the pathogenesis of emotional disorders, such as the dysfunction of Glu neurotransmissions in the cerebral cortex and marginal regions, which are closely related to depression [83, 84].

The cystine/Glu reverse transporter system (SystemXc−), a transmembrane amino acid transporter system in CNS cells, allows the exchange of the extracellular cystine with intracellular Glu and regulates the extracellular Glu concentration. Intracellular cystine uptake can be reduced to cysteine, which is an essential precursor for conversion to the antioxidant GSH [113–115]. The depolarization of presynaptic neurons and subsequent influx of Ca^2+^ can induce the release of Glu stored in vesicles into the synaptic cleft, where it binds to the corresponding target receptors [116, 117]. On the other hand, the free or excessive Glu is immediately removed and detoxified by the excitatory amino acid reuptake transporters (EAATs), thereby preventing the “Glu spillover effect” [83]. In this process, EAATs are the special transporter proteins found on the synaptic surface of neurons and glial cells [118, 119]. Releasing the Glu in large amounts can induce the excessive activation of NMDAR both inside and outside the synapse, which reduces the expression of NTF, such as BDNF, causing excitotoxicity, synaptic damage, and neuronal degeneration [13, 14, 120, 121].

Numerous studies showed that inflammation could affect the release and reuptake process of Glu [116, 120, 122]. For example, the activation of NF-κB signaling increased the IL-1β levels, which in turn promoted the expression of SystemXc− and increased the release of Glu to the outside of the synapse through the vesicular pathway [123, 124]. The inflammatory cytokines-induced OS could also play a similar role [120]. In addition, inflammatory factors can also promote the Glu release through other non-vesicular pathways, such as impairing the expression and function of EAATs in astrocytes, which resulted in reducing the reuptake and clearance of synaptic Glu [116, 125]. Other studies indicated that inflammatory factors could also reverse EAATs, thereby further increasing the levels of Glu in the synaptic cleft [126, 127].

#### Effect of neuroinflammation on HPA axis

The HPA axis, an important component of the neuroendocrine system, can maintain the homeostasis of the stress response system and internal environment, producing substances, such as adrenocorticotropin-releasing hormone (CRH), adrenocorticotropic hormone (ACTH), cortisol (CORT), and glucocorticoids (GC). The GC receptors (GRs) are widely distributed in the CNS, such as the hypothalamus, pituitary, adrenal gland, and cortex margin [128]. Under normal conditions, the binding of GC to GRs acts as a negative feedback inhibitor on the HPA axis, regulating its activation status. Under depression conditions, the expression of GRs decreases, which weakens the inhibition of the HPA axis, leading to the excessive activation of the HPA axis, thereby increasing the levels of CRH, ACTH, CORT, and GC in the patients with depression and affecting the secretion in vivo [85, 86].

Studies showed that sustained stress can also stimulate the release of CORT from the HPA axis; CORT can disrupt the balance of gut microbiota, causing intestinal permeability [129]. Furthermore, the CORT-induced increase in TDO enzyme activity can over-activate the Kyn pathway, which leads to decreasing the production of 5-HT [130]. CORT also plays an important role in regulating CNS-related activities, such as learning, memory, and emotion; the overproduction of CORT might alter the function of the hippocampus, prefrontal cortex (PFC), etc. [131]. Under normal conditions, GC is a powerful anti-inflammatory molecule and suppresses the synthesis and efficacy of cytokines, thereby blocking numerous inflammatory pathways [117]. However, high GC levels can trigger microglia-induced neuroinflammation and impair the integrity of neuronal membranes by interfering with the neuronal repair function of BDNF and promoting neurotoxicity and atrophy in the hippocampus [132–134].

Notably, inflammation can significantly affect the neuroendocrine function through the HPA axis [132]. The continuous production of proinflammatory cytokines, such as IL-6 [128], can increase the activation time of the HPA axis, increase GC synthesis, inhibit GRs and downregulate their function, cause negative feedback regulation disorder, and finally cause the disorder of GC level regulation [135]. Further studies suggested that proinflammatory cytokines could disrupt the signaling pathways, such as NF-κB, p38MAPK, and signal transducer and activator of transcription 5 signaling pathways, resulting in the impaired function of GRs [136, 137]. For example, IL-1α can inhibit the GRs-mediated gene transcription by activating the p38MAPK signaling pathway [138]. In addition, GR-β as an inactive form of GRs, can be activated by proinflammatory cytokines, thereby inhibiting the function of GRs [139].

#### Effect of neuroinflammation on NTF

NTF plays an important role in maintaining the function of the peripheral and CNS and provides relevant nutritional support for regulating emotional behavior in the nervous system [140]. BDNF, the most common type of NTF, can alter synaptic plasticity, increase synaptic connections, and promote long-term potentiation [141], which has a significant effect on neuronal morphology and physiology. Numerous studies showed that BDNF could produce antidepressant-like effects in the PFC and hippocampus. Interestingly, BDNF, acting on the ventral tegmental area (VTA)–nucleus accumbens (NAc) signaling pathway, can induce a depression-like phenotype [142]. Numerous recent studies indicated that BDNF and/or tropomyosin receptor kinase B (TrkB) signaling pathways could play important roles in the rapid antidepressant effects of ketamine [143]. For example, BDNF might trigger a mammalian target of the rapamycin protein (mTOR) signaling pathway, causing synaptogenesis [144, 145]. In addition, BDNF could also bind to TrkB receptors, activating other signaling pathways, such as phosphatidylinositol 3-kinase (PI3K)/Akt and MAPK signaling pathways, which play a role in mood disorders and depression [146–148]. Furthermore, an increase in the serum levels of glial-derived neurotrophic factor and transforming growth factor-β could improve depressive behavior [149].

Studies suggested that several inflammatory cytokines could downregulate the expression levels of BDNF and TrkB in the PFC and hippocampus and inhibit the phosphorylation of TrkB, thereby blocking the BDNF/TrkB signaling pathway and ultimately inducing the apoptosis of neurons [87]. In addition, inflammatory cytokines can also induce the hyperactivation of IDO and promote the Trp metabolism through the Kyn pathway, leading to the production of neurotoxic metabolites, such as 3‑HK and QA [93–95]. Among them, the stimulation of NMDAR by QA can cause the excitatory toxicity of Glu and signaling cascade, thereby reducing the BDNF expression and ultimately disrupting neurogenesis and synaptic plasticity [150, 151]. Notably, inflammatory mediators can also affect NFT3 and nerve growth factor to varying degrees [152].

## Correlations between depression and inflammation based on α7 nAChR

### Overview of α7 nAChR

The α7 nAChR, belonging to the Cys-loop receptor family, is a homopentamer ligand-gated ion channel assembled from five α7 subunits [153]. The five subunits of α7 nAChR are arranged in a pentagonal shape, forming an ion channel in the center, which mainly controls the flow of Na^+^, K^+^, and Ca^2+^ ions in and out of cells. Cys-loop receptors have three common structures, including the extracellular domain (ECD), transmembrane domain (TMD), and intracellular domain (ICD) [154]. The ECD contains the binding site for the agonist. The TMD has four hydrophobic areas designated M1, M2, M3, and M4, among which, M2 forms the inner lining of the ion channel and has an affinity for cations due to containing more acidic amino acid residues [155]. As compared to ECD and TMD, the ICD has more complex functions and plays important roles in the localization, transport, and assembly of receptors, thereby affecting the conductance and desensitization of channels and regulating downstream signaling pathways [156–158]. Consequently, ICD has been recognized as a potential target for drug design [159]. Recently, the Noviello CM team introduced the structure and dynamic interconversion process of α7 nAChR in three states, including resting, activated, and desensitized states, and reported an ECD component, which might be related to the relative permeability of the receptor [160]. Notably, X-ray crystallography and freeze electron microscopy could not analyze the specific structure of ICD due to the flexibility of its structure [161, 162]. Bondarenko et al. combined the experimental structure limitations of nuclear magnetic resonance and electron spin resonance spectroscopy with Rosetta calculation to determine the full-length ICD structure of human α7 nAChR [154].

As one of the most abundant subtypes in the human brain, α7 nAChR is enriched in the hippocampus, PFC, VTA, NAc, locus coeruleus, hypothalamus, dorsal raphe nucleus, and other brain regions, and is related to various CNS functions and diseases [163, 164]. Numerous studies indicated that α7 nAChR was expressed in different locations in neuronal cells, including presynaptic membrane, postsynaptic membrane, and perisynaptic [24]. In addition, α7 nAChR was also expressed in various non-neuronal cells, such as macrophages/monocytes [165], lymphocytes, DCs [166], microglia [167], astrocytes [168, 169], endothelial cells, bronchial epithelial cells, and vascular smooth muscle cells [153].

α7 nAChR is closely related to learning, memory, neuroprotection, synaptic plasticity, movement, attention, and anxiety [56, 170–173]. The ligand-binding sites of α7 nAChR mainly include agonist/antagonist-binding sites and allosteric modulator-binding sites, which are activated by agonists and positive allosteric modulators (PAMs), respectively. The binding of agonists to the extracellular ligand-binding domain of α7 nAChR can cause the rapid opening of central ion channels within milliseconds. Due to the high permeability of α7 nAChR to Ca^2+^, a large Ca^2+^ influx depolarizes the presynaptic membrane and promotes the fusion of neurotransmitter-containing vesicles and presynaptic membrane, thereby further increasing the release of neurotransmitters, such as ACh, NE, DA, Glu, and γ-aminobutyric acid. At the postsynaptic membrane, a large Ca^2+^ influx acts on the downstream Ca^2+^-sensitive kinases, which triggers a series of signal transduction processes [174, 175]. Although α7 nAChR is a ligand-gated ion channel, it can also increase the intracellular cyclic adenosine monophosphate (cAMP) levels through adenylate cyclase 1, a common signaling pathway for G protein-coupled receptors [24]. Thus, the activated α7 nAChRs have ionotropic and metabotropic functions in neurons and immune cells, including microglia, and are involved in regulating the Ca^2+^ influx, neurotransmitter release, and intercellular signal transduction [171, 176]. Moreover, the activated α7 nAChRs are closely related to autophagy, necrosis, transcription, apoptosis, and inflammatory processes in the body [170, 177, 178]. For instance, Hung et al. found that α7 nAChR could bind to amyloid-beta (Aβ), leading to its internalization into the cytoplasm and further inhibition of Aβ-induced neurotoxicity through autophagy. On the other hand, Lc3 and melatonin could enhance autophagy by increasing the expression of α7 nAChR, thereby showing neuroprotective effects. Further studies showed that in microglia, the enhanced autophagy, induced by the activation of α7 nAChR, was mediated by activating the AMPK–mTOR–p70S6K signaling pathway [179]. Interestingly, Hou et al. indicated that in cardiomyocytes, the activation of Janus kinase 2 (JAK2) and PI3K could mediate the α7 nAChR activation-induced enhanced autophagy [180]. Thus, in different organs or cells, α7 nAChR activation-induced autophagy enhancement might act through different signaling pathways. In addition, Hua et al. also showed that the α7 nAChR agonist PNU-282987 could decrease the activation of caspase-3, increase the expression of anti-apoptotic protein B-cell lymphoma-2, and exert anti-apoptotic effects in microglia [181]. Numerous recent studies reported that activating the α7 nAChR could induce anti-inflammatory effects, which might be an effective way to treat depression, Alzheimer’s disease, and other CNS diseases [24].

### Evidence of α7 nAChR associated with depression

Numerous studies have shown that the activated α7 nAChR plays an essential role in the pathogenesis of depression. For example, the α7 nAChR disorder, such as the depression-like phenotype in α7 knockout mice, can trigger depression [142]. α7 nAChR is encoded by *Chrna7*. Pu et al. showed that the composition of gut microbiota in mice with *Chrna7* knocked out mice was abnormal, such as a decrease in the abundance of *Muribaculum intestinale* and an increase in those of *Helicobacter ganmani* and *Lactobacillus animalis*, showing depression-like phenotype. Furthermore, as compared to the control mice, the fecal microbiota transplantation into *Chrna7* knockout mice resulted in systemic inflammation, downregulation of synaptophysin, and depression-like phenotype in the mice treated with an antibiotic mixture [182]. Zhang et al. also showed that the *α7 nAChR* knockout mice did not alter the BDNF/TrkB signaling pathway and synapsis in the hippocampus and PFC but increased the BDNF/TrkB signaling pathway in NAc, showing a depression-like phenotype. Interestingly, the bilateral infusion of TrkB antagonist ANA-12 with NAc could restore the increase in synapses in NAc and rapidly exert antidepressant effects, while fluoxetine could not show similar effects [142]. In contrast, antidepressant-like effects were exhibited in animals by agonism of α7 nAChR, such as α7 nAChR agonists DMXBA and PNU-282987 [183–186]. Further studies have shown that the activated α7 nAChR could mediate the release of DA and NE in the rat’s hippocampus and PFC [187, 188] but showed no effects on the uptake of 5-HT [189]. However, the α7 nAChR agonists have the disadvantages of insufficient selectivity, tendency to desensitize receptors, and lack of data related to clinical trials; these factors limit the application of α7 nAChR agonists in the treatment of depression [24, 174].

Therefore, studying the application of α7 nAChR PAMs in antidepressant treatment may be a promising direction. α7 nAChR PAMs were effective only in the presence of endogenous agonist ACh and could further enhance the agonistic effects of ACh on α7 nAChR. There are two types of α7 nAChR PAMs; type I PAMs do not affect receptors desensitization, while type II PAMs can delay receptors desensitization and reactivate desensitized receptors [190, 191]. Studies have shown that NS-1738 (type I PAMs), PNU-120596 (type II PAMs) and PAM-2 (type II PAMs) cannot exert significant antidepressant effects after 0.5 h or 1 h of administration, but can significantly improve depressive-like behavior after 7 days of administration, which still has some advantages over traditional antidepressants [163, 192–194]. Interestingly, PAM-2 induced more potent and durable antidepressant-like activity compared to NS-1738 and PNU-120596, indicating the importance of dosing cycle [163, 194]. In addition, Targowska-Duda et al. showed that PAM did not have a high affinity for human 5-HT, DA, and NE transporter proteins (< 1 μM) [163]. Notably, α7 nAChR PAMs can also specifically target α7 nAChR without affecting the physiological functions of other receptors, thereby showing fewer side effects than α7 nAChR agonists while exerting precise pharmacological effects. However, α7 nAChR PAMs also have the same disadvantage of lacking sufficient relevant clinical data [195, 196]. Therefore, the active state of α7 nAChR is closely related to depression, but requires extensive experiments to confirm. Table 1 lists the changes in depression-like behavior of animals when targeting α7 nAChR.

**Table 1:** Effect of targeted α7 nAChR on depressive-like behavior

Compound type	Compound name	Study subject	Intervention	Outcome	References
		Male C57BL/6	Knock-out α7 nAChR	Presence of depressive-like behavior	[142, 182]
α7 nAChR agonist	PNU282987	Male Wistar rats	FST was performed after 24 h of administration (0.5 mg/kg)	Significantly improve depressive-like behavior	[183]
		Male adult Sprague–Dawley rats	CUMS induced depression 4 weeks and FST was performed after 35 min of administration (1 mg/kg)	Significantly improve depressive-like behavior	[185]
		C57BL/6 mice	Aβ1-42-induced depression, and FST was performed after 10 days of administration (1 mg/kg)	Significantly improve depressive-like behavior	[186]
	DMXBA(GTS-21)	Male Kunming mice	CRS-induced depression, FST, TST, and SPT were performed after 11 days of continuous administration (4 mg/kg)	Significantly improve depressive-like behavior	[184]
Type I PAMs	NS-1738	Male Wistar rats	FST was performed after 24 h of administration (1 mg/kg)	Does not significantly improve depression-like behavior	[183]
		Male C57BL/6J mice	FST and TST were performed after 30 min, 7 days and 14 days of administration (1 mg/kg, 10 mg/kg), respectively	Depression-like behavior was significantly improved after 7 days of administration (1 mg/kg, 10 mg/kg)	[163]
Type II PAMs	PNU-120596	Male C57BL/6J mice	LPS was injected after 0.5 h of administration (4 mg/kg), and TST and SPT were performed after 26 h of LPS injection, and FST was performed after 28 h of LPS injection	LPS-induced depression-like behavior was blocked, but the antidepressant effect was reversed by MLA (3 mg/kg)	[192]
			LPS was injected after 0.5 h of administration (4 mg/kg), and FST was performed after 28 h of LPS injection	LPS-induced depression-like behavior was blocked, but the antidepressant effect was reversed by MLA (3 mg/kg)	[193]
			FST and TST were performed after 30 min, 7 days and 14 days of administration (1 mg/kg, 10 mg/kg), respectively	Depression-like behavior was significantly improved after 7 days of administration (1 mg/kg, 10 mg/kg)	[163]
	PAM-2	C57BL/6J mice	FST were performed after 1 h, 7 days, 14 days and 21 days of administration (1 mg/kg), respectively	Depression-like behavior was significantly improved after 7 days, 14 days, and 21 days of administration (1 mg/kg)	[194]
		Male C57BL	FST and TST were performed after 30 min, 7 days and 14 days of administration (0.5 mg/kg, 1 mg/kg), respectively	Depression-like behavior was significantly improved after 7 days and 14 days of administration (0.5 mg/kg, 1 mg/kg)	[163]

*FST* forced swimming test, *CUMS* chronic unpredictable mild stress, *Aβ1-42* amyloid-beta1-42, *CRS* chronic restraint stress, *TST* tail suspension test, *SPT* sucrose preference test, *PAMs* positive allosteric modulators, *LPS* lipopolysaccharide, *MLA* methylprednisolone citrate

### Activation of α7 nAChR-mediated CAP for antidepressant effect and its mechanism

In recent years, the activation of α7 nAChR-mediated CAP in anti-depression therapy has attracted researchers’ attention [24–26]. It is well-known that the vagus nerve, which connects the brain and surrounding organs, plays an important role in CAP. After stimulation, it can activate α7nAChR, which is closely related to the inhibition of NF-κB activation [56, 197, 198]. NF-κB is a transcription factor, which coordinates the inflammatory response and regulates the expression levels of inflammatory genes [199]. The NF-κB-binding inhibitors of NF-κB (IκB) are normally present in the cytoplasm. Studies have shown that vagal stimulation can upregulate the α7 nAChR expression in hippocampal microglia and exert anti-inflammatory effects by inhibiting the nuclear translocation of NF-κB and phosphorylation of p65. However, the vagus nerve stimulation could not exert similar anti-inflammatory effects after vagotomy or injection of α7 nAChR antagonists or using α7nAChR(−/−) rats [198, 200]. Thus, the α7 nAChR/NF-κB signaling pathway might play a crucial role in the CAP. However, the mechanism of α7 nAChR, affecting the upstream and downstream pathways of NF-κB, requires further investigation.

**Fig. 2:**
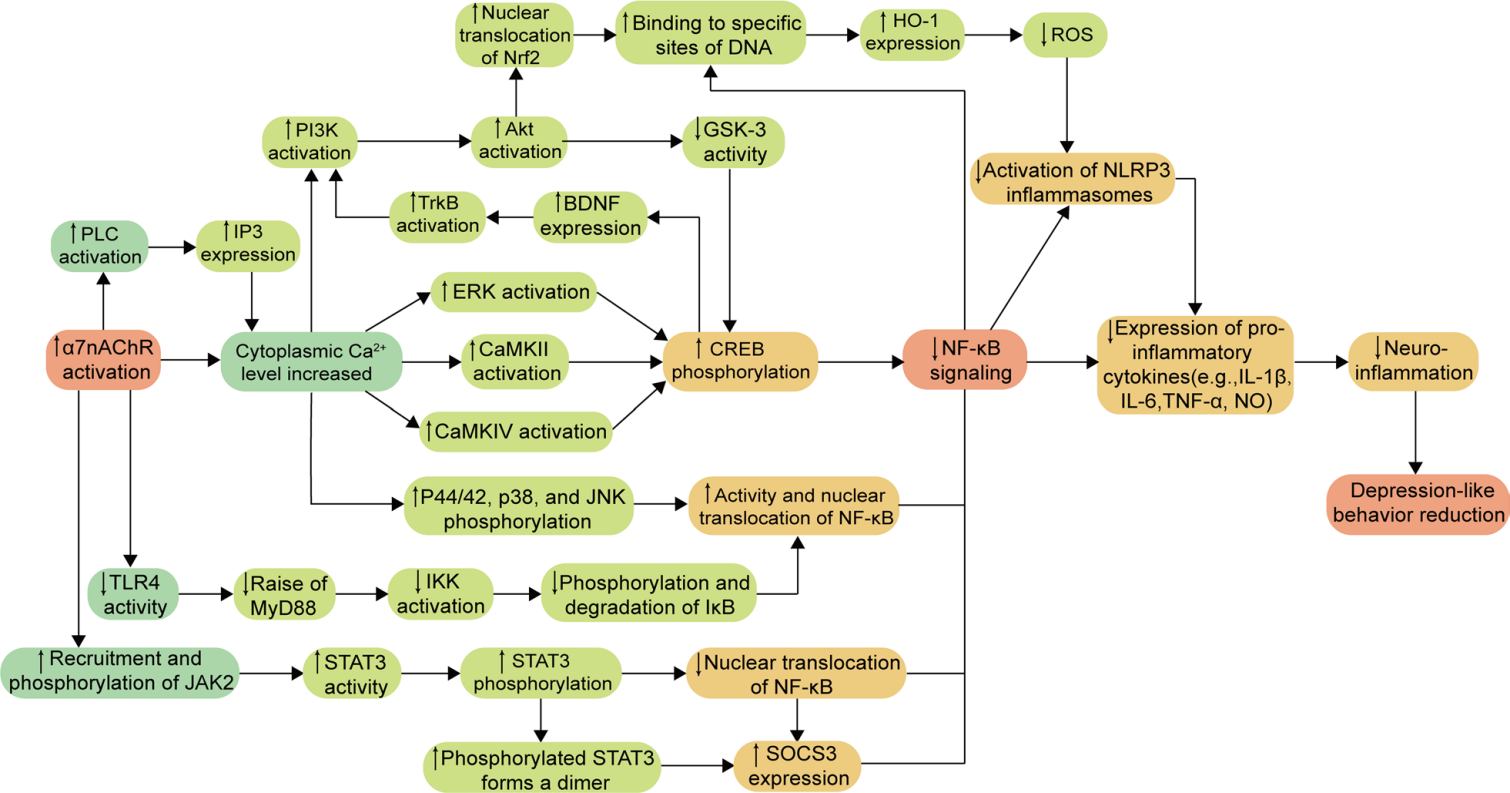
Molecular mechanisms of activation of α7 nAChR-mediated CAP. The activation of α7 nAChR could inhibit the expression of NF-κB through TLR4/NF-κB/NLRP3, JAK2/STAT3/NF-κB and Ca^2+^-related signaling pathways, reduce the production of inflammatory cytokines, reduce neuroinflammation, and finally play an antidepressant role. ↑: upregulate, ↓: downregulate, *TLR4* Toll-like receptors 4, *MyD88* myeloid differentiation factor 88, *IKK* inhibitor of kappa B kinase, *IκB* inhibitor of NF-κB, *JAK2* Janus Kinase 2, *STAT3* signal transduction and transcription activator 3, *SOCS3* suppressor of cytokine signaling 3, *NF-κB* nuclear factors-kappa B, *PLC* phospholipase C, *IP3* inositol 1,4,5-triphosphate, *PI3K* phosphatidylinositol 3-kinase, *Akt* protein kinase B, *GSK-3* glycogen synthase kinase 3, *BDNF* brain-derived neurotrophic factor, *TrkB* tropomyosin receptor kinase B, *ERK* extracellular signal-regulated kinase, *CaMKII* Ca^2+^/calmodulin-dependent protein kinase II, *CaMKIV* Ca^2+^/calmodulin-dependent protein kinase IV, *JNK* c-Jun N-terminal kinase, *Nrf2* nuclear transcription factor E2-related factor, *HO-1* heme oxygenase-1, *ROS* reactive oxygen species, *CREB* cAMP-response element binding protein, *NLRP3* NOD-like receptor protein 3, *IL-1β* interleukin-1β, *IL-6* interleukin-6, *TNF-α* tumor necrosis factor-α, *NO* nitric oxide

An in-depth study revealed that the activation of α7 nAChR exerted anti-inflammatory effects through the TLR4/NF-κB/NLRP3, JAK2/STAT3/NF-κB, and Ca^2+^-related signaling pathways [184, 192, 201–207]. In addition, activating the α7 nAChR can activate chronic stress-induced neuroinflammation to promote Tregs cell function. As a subset of CD4^+^ T cells, Tregs can protect the BBB, inhibit the infiltration of peripheral inflammatory cells and factors into the brain, and play an important role in maintaining immune homeostasis [208, 209]. For instance, Zhao et al. demonstrated that the treatment with α7 nAChR agonist DMXBA could reverse the chronic stress-induced increase in Tregs cells, thereby limiting the inflammatory response in the brain and attenuating the depression-like behavior in chronic restraint stress (CRS) mice [184]. The specific molecular mechanisms of α7 nAChR’s anti-inflammatory effects around NF-κB are summarized in the following sections (Fig. 2).

#### Activation of α7 nAChR triggers TLR4/NF-κB/NLRP3

TLRs are significant receptors on the surface of microglia and play important roles in neuroinflammation-mediating signaling pathways [210]. TLRs can be activated by recognizing PAMP or DAMP, and the activated TLR4 can recruit the adaptor protein myeloid differentiation factor 88 (MyD88) [57, 58]. The stimulation by MyD88 and cytokines, such as TNF-α, can activate the inhibitor of kappa B kinase, which in turn phosphorylates and subsequently degrade IκB, thereby promoting the activation of NF-κB and its entry into the nucleus and increasing the transcriptional expression of inflammasome components and pro-IL-1β [43, 211]. NLRP3 inflammasome is a multi-protein assembly, which consists of NLRP3 (cytoplasmic sensor molecule), adapter protein caspase activation recruitment domain, and effector protein pro-caspase-1 [212]. Studies have shown that the activation of NLRP3 inflammasome is closely related to NF-κB [59] and generally requires the initiation and activation of two signals, including the initiation signal and activation signal. The initiation signal involves the activation of NF-κB induced by the activation of TLR, while in the activation signal, the sustained stimulation induces the inflammasome components to assemble into complete NLRP3 inflammasome and promotes the conversion of pro-caspase-1 into activated caspase-1, which subsequently cleaves the pro-IL-1β and pro-IL-18 into active forms, ultimately leading to an inflammatory response and induction of cytotoxicity [42, 212, 213]. In addition, studies have also shown that ROS can activate the NLRP3 inflammasome [214, 215].

The selective α7 nAChR partial agonist DMXBA could alleviate chronic stress-induced activation of the TLR4 signaling pathway and exert antidepressant-like behavior in mice; however, this effect was reversed after pretreatment with the selective α7 nAChR antagonist α-bungarotoxin (α-BGT), indicating that the protective effects of DMXBA were α7 nAChR-dependent. Interestingly, using α-BGT at the dose (1 μg/kg/d) did not reverse the inhibitory effects of DMXBA on the activation of microglia [184]. Deng et al. showed that the activation of α7 nAChR could significantly inhibit the expression of NLRP3 inflammasome; this result was consistent with those of a study by Fu [201, 202]. Furthermore, activating the α7 nAChR could also attenuate OS [216]. Therefore, the activation of α7 nAChR might exert anti-neuroinflammatory effects by inhibiting the TLR4/NF-κB/NLRP3 signaling pathway and reducing OS.

#### Activation of α7 nAChR triggers JAK2/STAT3/NF-κB

JAK2 is widely distributed in the cytoplasm of somatic cells and is important in activating immune cells. STAT3 is an essential transcription factor, which regulates the expression of downstream target genes associated with the differentiation and apoptosis of cells [217]. JAK2/STAT3 is the most crucial signaling pathway in the JAK–STAT family and is closely related to inflammation [218]. The recruited and phosphorylated JAK2 can activate STAT3 to induce the phosphorylation of STAT3, which then prevents the nuclear translocation of NF-κB [170]. In addition, the phosphorylated STAT3 can readily form dimers to enter the nucleus and bind to DNA, thereby positively regulating the transcription of suppressor of cytokine signaling 3, which leads to inhibiting NF-κB activation and reducing the production of inflammatory cytokines, such as TNF-α and IL-1β [170].

Numerous studies showed that the activated α7 nAChR could regulate other signal transduction pathways by promoting the JAK2/STAT3 signaling pathway and regulating the gene transcription in immune cells independent of ion influx [176, 192, 203]. For example, blocking the JAK2 phosphorylation using AG490 attenuated the inflammatory regulatory effects of α7 nAChR agonists; inhibiting the STAT3 phosphorylation also showed a similar effect [205]. Zhao et al. showed that the treatment with the α7 nAChR agonist DMXBA could significantly reverse the CRS-induced downregulation of STAT3 in the hippocampal nucleus, thereby restoring the central cholinergic signaling function [184]. Therefore, the activation of α7 nAChR might attenuate the inflammatory response by promoting the JAK2/STAT3 signaling pathway and ultimately inhibiting the activation of NF-κB.

#### Activation of α7 nAChR triggers Ca^2+^-related signaling pathway

The PI3K/Akt and extracellular signal-regulated kinase (ERK) signaling pathways play an important role in cell proliferation and maturation [219]. Ca^2+^/calmodulin-dependent protein kinase II (CaMKII) is associated with the enhancement of learning and memory as well as synaptic remodeling, while CaMKIV is involved in regulating the growth of DCs in cortical and hippocampal neurons [220–222]. A large Ca^2+^ influx can activate the PI3K/Akt, ERK, CaMKII, and CaMKIV signaling pathways [223–226], thereby increasing the phosphorylation of cAMP-response element binding protein (CREB) at residue Serine-133 and its subsequent BDNF expression [227–229]. The phosphorylated CREB can compete with CREB-binding protein to bind with NF-κB; this process is affected by the activity of glycogen synthase kinase-3beta (GSK-3β) and inhibits the transcription of NF-κB, thereby playing an anti-inflammatory role and promoting adult hippocampal neurogenesis [230–233]. Among them, the activated PI3K/Akt signaling pathway can facilitate the nuclear translocation of nuclear transcription factor E2-related factor (Nrf2), which increases the binding of Nrf2 to electrophilic response elements (EpRE) or antioxidant response elements at DNA-specific sites as well as the expression levels of antioxidant genes, such as heme oxidase-1 [234], thereby inhibiting the degree of OS and ultimately reducing the production of the proinflammatory cytokines TNF-α and IL-1β. In addition, studies have shown that PI3K/Akt can inhibit the GSK-3 activity, while GSK-3 can inhibit the CREB signaling pathway [235]. Notably, some studies indicated possible interactions between NF-κB and Nrf2; the NF-κB subunit p65 can negatively regulate Nrf2 and inhibit its interaction with EpRE [236]. Furthermore, an increase in the intracellular Ca^2+^ levels can also inhibit the activation of NF-κB and subsequent nuclear translocation by suppressing the phosphorylation of neuroinflammation-associated p44/42, p38, and c-Jun N-terminal kinase [237, 238]. This can ultimately reduce the production of inflammatory mediators, such as IL-6, TNF-α, and NO [239, 240].

Activating the α7 nAChR can trigger a large Ca^2+^ influx [206]. Moreover, the activated α7 nAChR in mice microglia can activate phospholipase C via Gαq, producing 1,4,5-triphosphate (IP3), which can bind to the IP3 receptor on the endoplasmic reticulum, inducing the release of Ca^2+^ from the endoplasmic reticulum [207]. Recently, Morioka et al. proposed that activating the α7 nAChR-mediated IP3 and Ca^2+^/CaMKII signaling pathways upregulated the expression of Glu/aspartate transporter protein and increased Glu uptake [24]. Therefore, the activation of α7 nAChR can trigger the process of massive Ca^2+^ influx and release from the endoplasmic reticulum, which might increase intracellular Ca^2+^ levels and promote a series of signaling pathways, ultimately inhibiting the inflammatory response.

## Conclusions and prospects

This review article summarized the relationship between inflammation and depression as well as several pathways of neuroinflammation affecting depression, such as neuroinflammation can affect the synaptic availability of monoamine neurotransmitters and Glu, increase the activation time of the HPA axis, regulate the BDNF/TrkB signaling pathway in various brain regions. This, in turn, aggravates neurotoxicity and damages neurogenesis and synaptic plasticity, which finally induces neuronal apoptosis and triggers depression and other related psychiatric diseases. This review article also highlighted the crucial role of α7 nAChR in the CAP and suggested that its activation might exert anti-inflammatory effects by promoting Tregs function and through TLR4/NF-κB/NLRP3, JAK2/STAT3/NF-κB, and Ca^2+^-related signaling pathways, thereby alleviating depression-like behavior.

Depression is the result of multiple mechanisms; the recent drugs developed based on the hypothesis of monoamine neurotransmitters could not achieve favorable clinical outcomes. However, the importance of the inflammation hypothesis in the occurrence of depression has gradually been recognized, especially the activation of α7 nAChR-mediated CAP. Notably, the BBB should also be considered as a factor in developing the corresponding PAMs due to the large distribution of α7 nAChR within and outside the CNS. Therefore, based on the idea of activating the α7 nAChR-mediated CAP to exert antidepressant effects, developing the α7 nAChR type II PAMs, capable of passing through the BBB, might be a current research direction for developing new antidepressant drugs. Furthermore, the results should be confirmed in a large number of subsequent preclinical and clinical trials.

## Data Availability

Not applicable.

## Abbreviations

α7 nAChR: Alpha7 nicotinic acetylcholine receptor
CAP: Cholinergic anti-inflammatory pathway
5-HT: 5-Hydroxytryptamine
DA: Dopamine
NE: Norepinephrine
Ach: Acetylcholine
AchE: Acetylcholinesterase
CNS: Central nervous system
IL-6: Interleukin-6
IL-1β: Interleukin-1beta
TNF: Tumor necrosis factor
IL-4: Interleukin-4
TLRs: Toll-like receptors
DCs: Dendritic cells
PAMP: Pathogen-associated molecular pattern
DAMP: Damage-associated molecular pattern
NF-κB: Nuclear factors-kappa B
MAPK: Mitogen-activated protein kinase
IL-1: Interleukin-1
IL-18: Interleukin-18
BBB: Blood–brain barrier
TNF-α: Tumor necrosis factor alpha
NO: Nitric oxide
NLRP3: NOD-like receptor protein 3
BDNF: Brain-derived neurotrophic factor
Cx: Connexin
OS: Oxidative stress
ROS: Reactive oxygen species
GSH: Glutathione
NOS: Nitric oxide synthase
iNOS: Inducible NOS
Glu: Glutamic acid
HPA: Hypothalamus pituitary adrenal
NTF: Neurotrophic factors
BH4: Tetrahydrobiopterin
Trp: Tryptophan
Kyn: Kynurenine
MAO: Monoamine oxidase
IDO: Indoleamine 2,3-dioxygenase
TDO: Tryptophan 2,3-dioxygenase
3‑HK: 3-Hydroxykynurenine
Kynu: Kynureninase
3-HAA: 3-Hydroxy anthranilic acid
KAT: Kynurenine aminotransferase
QA: Quinolinic acid
Kyna: Kynurenic acid
NMDAR: *N*-Methyl-d-aspartic acid receptor
VMAT2: Monoamine transporter protein 2
DRD: Dopamine receptors
SystemXc−: Cystine/Glu reverse transporter system
GSH: Glutathione
EAATs: Excitatory amino acid reuptake transporters
CRH: Adrenocorticotropin-releasing hormone
ACTH: Adrenocorticotropic hormone
CORT: Cortisol
GC: Glucocorticoids
GRs: GC receptors
PFC: Prefrontal cortex
VTA: Ventral tegmental area
NAc: Nucleus accumbens
TrkB: Tropomyosin receptor kinase B
mTOR: Mammalian target of the rapamycin protein
PI3K: Phosphatidylinositol 3-kinase
ECD: Extracellular domain
TMD: Transmembrane domain
ICD: Intracellular domain
PAMs: Positive allosteric modulators
cAMP: Cyclic adenosine monophosphate
Aβ: Amyloid-beta
JAK2: Janus kinase 2
IκB: Inhibitors of NF-κB
CRS: Chronic restraint stress
MyD88: Myeloid differentiation factor 88
α-BGT: α-Bungarotoxin
ERK: Extracellular signal-regulated kinase
CaMK: Ca^2+^/calmodulin-dependent protein kinase
CREB: CAMP-response element binding protein
GSK-3β: Glycogen synthase kinase-3beta
Nrf2: Nuclear transcription factor E2-related factor
EpRE: Electrophilic response elements
IP3: 1,4,5-Triphosphate
